# Cancer-Associated Fibroblasts Regulate the Plasticity of Breast Cancer Stemness through the Production of Leukemia Inhibitory Factor

**DOI:** 10.3390/life11121298

**Published:** 2021-11-26

**Authors:** Nazanin Vaziri, Laleh Shariati, Ali Zarrabi, Ali Farazmand, Shaghayegh Haghjooy Javanmard

**Affiliations:** 1 Department of Cell and Molecular Biology, Kish International Campus, University of Tehran, Kish 7941639982, Iran; vaziri.nazanin@yahoo.com; 2 Cancer Prevention Research, Isfahan University of Medical Sciences, Isfahan 8158388994, Iran; L.shariati@amt.mui.ac.ir; 3 Department of Biomaterials, Tissue Engineering and Nanotechnology, School of Advanced Medical Technologies, Isfahan University of Medical Sciences, Isfahan 8158388994, Iran; 4 Department of Biomedical Engineering, Faculty of Engineering and Natural Sciences, Istinye University, Istanbul 34396, Turkey; ali.zarrabi@istinye.edu.tr; 5 Department of Cell and Molecular Biology, School of Biology, University College of Science, University of Tehran, Tehran 1417614411, Iran; 6 Applied Physiology Research Center, Isfahan Cardiovascular Research Institute, Isfahan University of Medical Sciences, Isfahan 8158388994, Iran

**Keywords:** breast cancer, leukemia inhibitory factor, cancer stem cell, cancer-associated fibroblasts, LIF/LIFR signaling pathway

## Abstract

**Simple Summary:**

Cancer-associated fibroblasts (CAFs; as components of cancer stroma) use different signaling pathways to promote tumor progression and growth, invasion, and metastasis in diverse cancers. They have crosstalk with cancer cells during tumor progression. To investigate this crosstalk, in this study, a coculture system of CAF cells isolated from breast cancer patients with breast cancer cells was used to investigate leukemia inhibitory factor (LIF) production from CAFs. LIF is a signaling molecule that activates distinct signaling pathways through which cell cycle progression, cell death, adhesion, migration, and tumorigenesis are regulated.

**Abstract:**

Leukemia inhibitory factor (LIF), as a member of the interleukin-6 cytokine family, plays a complex role in solid tumors. However, the effect of LIF as a tumor microenvironment factor on plasticity control in breast cancer remains largely unknown. In this study, an in vitro investigation is conducted to determine the crosstalk between breast cancer cells and fibroblasts. Based on the results, cancer-associated fibroblasts are producers of LIF in the cocultivation system with breast cancer cells. Treatment with the CAF-CM and human LIF protein significantly promoted stemness through the dedifferentiation process and regaining of stem-cell-like properties. In addition, the results indicate that activation of LIFR signaling in breast cancer cells in the existence of CAF-secreted LIF can induce *Nanog* and *Oct4* expression and increase breast cancer stem cell markers CD24−/CD44+. In contrast, suppression of the LIF receptor by human LIF receptor inhibition antibody decreased the cancer stem cell markers. We found that LIF was frequently overexpressed by CAFs and that LIF expression is necessary for dedifferentiation of breast cancer cell phenotype and regaining of cancer stem cell properties. Our results suggest that targeting LIF/LIFR signaling might be a potent therapeutic strategy for breast cancer and the prevention of tumor recurrence.

## 1. Introduction

Breast cancer is the second-largest cause of cancer death following lung cancer in the United States. In 2021, 43,600 people died from breast cancer, along with 281,550 newly reported cases [1]. These numbers are down due to advances in prevention, surgical resection, and assistant treatments. The main reason for death in breast cancer is metastasis to vital organs, including lung, bone, and brain. In light of medical interventions, chemotherapy, and radiation treatment, many cancers are controlled; nevertheless, metastasis remains a problem [2,3].

Cancer stroma plays a critical role in how tumors progress in breast cancer and other malignancies and consists of six components: basement membrane, inflammatory cells, capillaries, immune cells, extracellular matrix, and cancer-associated fibroblasts (CAFs) or activated fibroblasts. Typically, distinguishing CAFs from other fibroblasts is based on the expression of two biomarkers: fibroblast activation protein alpha (FAP) and alpha-smooth muscle actin (α-SMA) [4]. α-SMA is now one of the most commonly used biomarkers to identify CAF populations and has been discovered to be a significant predictive factor in cancer patients. α-SMA-positive fibroblasts have been associated with a lower overall survival rate in breast cancer [5]. CAFs use different signaling pathways to promote tumor progression and growth, invasion, and metastasis in diverse cancers [6]. They have crosstalk with cancer cells during tumor progression [6]. Several studies have evaluated the role of CAFs in breast cancer and suggested that CAFs promote tumor proliferation [7], invasion, metastasis [8], epithelial–mesenchymal transition (EMT) [9], and angiogenesis [10].

In addition, the tumor microenvironment is composed of a small population of cells named cancer stem cells (CSCs). These stem cells are tumorigenic stem-like cells with the ability to promote asymmetric division, self-renewal, multipotency, differentiation into specialized cell types, and development into cancer [11]. The phenotype of ‘cancer stemness’ could be the driving force in the development of cancer and CSCs may cause most malignant tumors that with tumor niche contribute to cancer metastasis, drug resistance, and recurrence [11,12]. Thus, therapeutic strategies targeting the cancer microenvironment or CSCs hold promise for cancer treatment [13]. More studies exhibit that CSCs are present in different solid tumors, including breast cancer, which is characterized by a CD24 −/CD44+ cell surface marker [14,15]. The tumor stroma factors are observed to have a significant role in the growth and survival of cancer cells and signaling pathways; however, their effect on cancer cell plasticity remains unknown [16].

Alteration in the expression of signaling molecules plays an important role in the activation of the PI3K/AKT and JUN/MAPK pathways, leading to the development of breast cancer [17]. One of the signaling molecules is leukemia inhibitory factor (LIF), with a molecular weight ranging from 38 to 67 kDa, which is a pleiotropic cytokine and belongs to the interleukin-6 cytokine superfamily [18]. The biological functions of LIF are mediated by binding LIF to a complex receptor that comprises the LIF receptor (LIFR) and the glycoprotein gp130 subunit, which activates distinct signaling pathways JAK/STAT3, MAPK, PI3K/AKT, and ERK1/2 [18,19]. Activation of these signaling pathways by LIF regulates cell cycle progression, cell death, adhesion, migration, and tumorigenesis [20].

Human LIF is a multifunctional protein with a wide array of actions, including the regulation of some hematopoietic cells [21], development of platelets, regulation of self-renewal function of embryonic stem cells and maintaining their pluripotency [22], implantation of the developing embryos [23], growing of the embryonic stem cells of mouse, bone development through stimulating osteoblast differentiation [24], production of adrenocorticotropic hormone, neuronal development [25], and proliferation of muscle satellite cells [26,27].

Through activation of the AKT-mTOR signaling pathway, LIF induces breast cancer cell proliferation, invasion, and metastasis [27]. Breast cancer cells T47D and MDA-MB-231, treated with LIF injected into mice, induced lung and neck metastasis compared to the control [28]. In mouse breast tumors, a high level of LIF expression and activated STAT3 signaling have been observed, resulting in increased tumor cell viability [29]. Through STAT3 signaling activation, LIF enhances miR-21 production, and miR-21 induces epithelial–mesenchymal transition [30]. Activation of JAK1-STAT3 signaling decreases drug responsiveness in breast cancer by upregulating LIFR [31]. These findings suggest that blocking LIF/LIFR signaling could be a possible breast cancer treatment.

In this study, a coculture system of CAF cells isolated from breast cancer patients with breast cancer cells was used to investigate LIF production from CAFs. Furthermore, the effect of LIF protein on the dedifferentiation process of breast cancer cells to breast cancer stem cells through the LIF/LIFR pathway is studied. As far as we investigated, this is the first attempt to study the regulatory influence of CAFs on breast cancer stemness through LIF function.

## 2. Materials and Methods

### 2.1. Materials

The following materials were used in this study: RPMI 1640 media (Bio Idea, Tehran, Iran, Cat. No.: BI-1006), fetal bovine serum (FBS) (Bio Idea, Tehran, Iran, Cat. No.: BI-1201), phosphate-buffered saline (PBS; Bio Idea, Tehran, Iran, Cat. No.: BI-1401), DMEM (Bio Idea, Tehran, Iran, Cat. No.: BI-1012), anti-α-SMA antibody (1/500, Abcam (Cambridge, UK), Cat. No.: ab5694), HRP-conjugated secondary goat anti-rabbit IgG (1/2000, Abcam, Cat. No.: ab205718), 24-well 8.0 μm pore size Cell Culture plate (Corning, Cat. No.: 353097), human LIF protein (Sino Biological, Beijing, China, Cat. No.: LIF 14890-HNAH), LIF receptor inhibitor antibody (Millipore (Burlington, MA, USA), Cat. No.: MABD150), FITC-conjugated mouse monoclonal antibody against human CD44 (5/100, Biolegend(San Diego, CA, USA), Cat. No.: 338803), PE-conjugated mouse monoclonal antibody against human CD24 (5/100, Biolegend, (San Diego, CA, USA), Cat. No.: 311105), Total RNA Kit (Yekta Tajhiz, Tehran, Iran, Cat. No.: YT9080), and cDNA synthesis kit (BioFact, Daejeon, Korea, Cat. No.: BR441-096).

### 2.2. Breast Cancer Cell Lines

The human breast cancer cell lines MDA-MB-231 (C578, RRID: CVCL_0062) and MCF7 (C135, RRID: CVCL_0031) were obtained from the Pasteur Institute of Iran (Pasteur Institute of Iran, Tehran, Iran). Breast cancer cell lines were cultured and maintained in RPMI 1640 media supplemented with 10% fetal bovine serum (FBS) (Bio Idea, Tehran, Iran, Cat. No.: BI-1201) at 37 °C under 20% O_2_ and 5% CO_2_.

### 2.3. Isolation and Verification of CAFs

CAFs were gathered from six freshly discarded breast tumor tissues belonging to grade 3 breast cancer patients who underwent resection of their breast tumor at the Isfahan Seyed-al-Shohada Hospital. Written informed consent was obtained from all patients. Tumor tissues were harvested within 30 min following resection and were placed in DMEM containing 10% FBS for immediate transportation on ice to the laboratory. Tissues were washed with phosphate-buffered saline (PBS) and minced into small pieces to prepare cell suspensions. The cell suspension was centrifuged at 1000 rpm for 4 min, resuspended in the fresh DMEM (Bio Idea) with 10% FBS, seeded into the 100-mm tissue-culture plates, and incubated at 37 °C in a humidified atmosphere containing 20% O_2_ and 5% CO_2_. The media was changed three times a week. CAFs were grown and used during six passages in all experiments.

### 2.4. Immunocytochemistry (ICC)

Alpha-actin smooth muscle (α-SMA) is known as a specific marker widely expressed in fibroblasts [6]. After one week when CAFs were completely extracted from the tissue, they were cultured in a 25 cm² flask for one more week and then were stained on Day 14. The anti-α-SMA antibody (1/500, Abcam (Cambridge, UK), Cat. No.: ab5694) and HRP-conjugated secondary goat anti-rabbit IgG (1/2000, Abcam (Cambridge, UK), Cat. No.: ab205718) were used to identify CAFs by immunocytochemistry based on the company instructions on Day 14.

### 2.5. Collection of Conditioned Media (CM)

To collect the CM of CAFs, cancer-associated fibroblasts were cultured to reach 80–90% confluency in 60 mm dishes in DMEM with 10% FBS. After washing three times with PBS, the cells were cultured for an additional 24, 48, and 72 h in 6 mL of the culture media without FBS. Later, the culture supernatant was harvested and centrifuged at 1600 rpm for 4 min, and then a 0.22 μm filter was used to filter the supernatant. The obtained media were diluted in RPMI 1640 with 10% FBS (ratio: ½) and were used as the CAF-CM.

### 2.6. Evaluation of Cancer Cells’ Effect on LIF Expression of CAFs

The 24-well 8.0 μm pore size Cell Culture plates (Corning, NY, USA), Cat. No.: 353097) were used for the cocultivation system. About 1 × 10^5^ CAFs were seeded into the lower chamber of each plate. Then, after 24 h, 2 × 10^4^ MCF7 or MDA-MB-231 cells were seeded into the upper chamber in test groups, and then the expression of LIF was measured by quantitative real-time PCR (qRT-PCR) analysis after 24, 48, and 72 h.

### 2.7. Evaluation of Cancer Cells’ Stemness after Treatments

#### 2.7.1. Cancer Cell Treatment with CAF-CM

About 5 × 10^4^ cancer cells (MDA-MB-231 and MCF7) were seeded into a 12-well plate among the test and control groups for 24 h, followed by the addition of CAF-CM to the plate. After 4, 6, and 10 days, the cells were harvested and analyzed by flow cytometry for CD24/CD44 as cancer stem cell markers. In addition, cells on Day 10 were collected to assess the expression of breast cancer stem cell markers *Nanog* and *Oct4* using qRT-PCR.

#### 2.7.2. Cancer Cell Treatment with LIF

To analyze the functional role of LIF, about 5 × 10^4^ cancer cells (MDA-MB-231 and MCF7) were seeded into a 12-well plate among the test and control groups. After 24 h, human LIF was obtained from Sino Biological (Cat. No.: LIF 14890-HNAH) and administered to cells in a concentration of 25 ng/mL, twice a week. After 3, 7, 10, and 14 days, the cells were evaluated for CD24 and CD44 markers. Additionally, cells on Day 14 were assessed for *Nanog* and *Oct4* expression and compared to control cells.

#### 2.7.3. Cancer Cell Treatment with LIF Receptor Inhibitor Antibody

About 5 × 10^4^ cancer cells (MDA-MB-231 and MCF7) were seeded into a 12-well plate for 24 h, and then 1 µg/mL human LIF receptor inhibitor antibody was added to the plate. After 2 h, human LIF protein was added to the media. This process was repeated in two-day intervals. After 4 and 7 days, cells were collected for the flow cytometry to analyze CD24/CD44 expression. In addition, cells on Day 7 were collected for the qRT-PCR to assess *Nanog* and *Oct4* expression. The experiment was also repeated by adding the CAF-CM instead of human LIF protein.

### 2.8. Flow Cytometry

The expression of cancer stem cell markers CD24/CD44 was determined by direct immunofluorescence staining with FITC-conjugated mouse monoclonal antibody against human CD44 (5/100, Biolegend, (San Diego, CA, USA), Cat. No.: 338803) and PE-conjugated mouse monoclonal antibody against human CD24 (5/100, Biolegend, (San Diego, CA, USA), Cat. No.: 311105). About 5 × 10^5^ cells were suspended in 200 µL of PBS and were incubated at 4 °C for 30 min after addition of both antibodies. After washing with PBS, cells were analyzed by flow cytometry (BD, San Jose, CA, USA). The unstained control was used to determine threshold values.

### 2.9. Quantitative Real-Time PCR

Total RNA was isolated from cells by using the Total RNA Kit (YektaTajhiz, Tehran, Iran, Cat. No.: YT9080) and reverse-transcribed into cDNA using cDNA synthesis kit (BioFact, Daejeon, Korea, Cat. No.: BR441-096) according to the manufacturer’s instructions. Produced cDNAs were used as templates in qRT-PCR. The primer sequences for *GAPDH*, *Oct4*, *Nanog,* and *LIF* are listed in Table 1. The cycling conditions were as follows: initial denaturation at 95 °C for 10 min, 40 cycles of denaturation at 95 °C for 15 s, followed by annealing and extension at 60 °C for 1 min.

### 2.10. Statistical Analyses

Statistical analyses were performed by using GraphPad Prism 4.0 software (San Diego, CA, USA). To compare the differences between the two groups, Student’s *t*-tests were applied. To compare multiple groups, one-way ANOVA methods with Tukey’s post hoc correlations were used. All results were shown as the mean ± SD, and values of *p* < 0.05 were defined as statistically significant.

## 3. Results

### 3.1. Evaluation of α-SMA Markers’ Exhibition by Isolated Fibroblast

**Figure 1:**
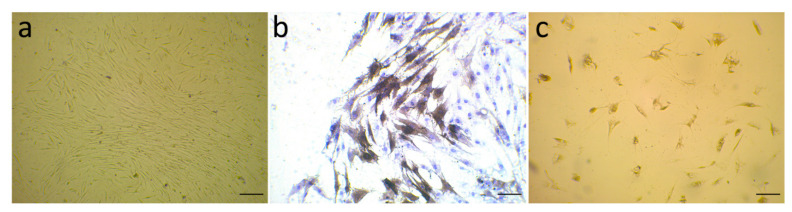
(**a**) Isolated CAFs were visualized under a microscope and were identified by irregular spindle-shaped and network structure after two weeks; (**b**) immunocytochemistry (ICC) labeling of α-smooth muscle actin (α-SMA) on Day 14 has confirmed CAF cells; (**c**) isolated normal fibroblasts as a control. Scale bars (lower right) = 100 μm.

The isolated stromal cells from primary breast tissues are a combination of different cell types, the main kind of which is the fibroblast cell line. The stromal fibroblasts from six human breast cancer tissues were isolated and cultured for 14 days and then stained to determine the expression of α-SMA, as a marker of activated myofibroblasts, on them. The purity of CAFs was verified by cell morphology (under a Leica microscope (Wetzlar, Germany) equipped with a Leica camera (DFC450)) and immunocytochemistry. Figure 1a represents the typical morphology of CAFs, Figure 1b shows the activated myofibroblast marker α-SMA expressed on isolated CAFs, and Figure 1c exhibits normal fibroblasts as a control. The α-SMA-positive cells in the cytoplasm are shown in brown color, and CAFs strongly expressed α-SMA.

### 3.2. Expression of LIF in Fibroblasts in the Presence of Breast Cancer Cells

**Figure 2:**
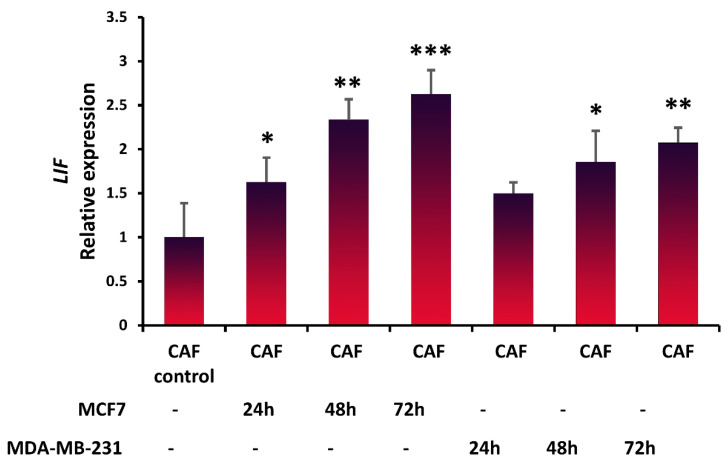
Breast cancer cells stimulate LIF expression in CAFs. Breast cancer cells were cocultured with CAFs, and after 24, 48, and 72 h, the quantitative real-time PCR analysis confirmed that LIF expression is enhanced. Representative graphs of 3 independent experiments using the same CAF-CM are shown. The data show the mean ± S.D. and were analyzed using one-way ANOVA with Tukey’s post hoc correlations; * *p* < 0.05, ** *p* < 0.01, and *** *p* < 0.001.

To study whether breast cancer cells stimulate CAFs to produce LIF, LIF expression was evaluated in CAFs after coculturing with breast cancer cell lines by performing qRT-PCR analysis. MCF7 significantly increased the expression of LIF in the CAFs (*p* < 0.05) after 24 h (Figure 2); however, the amount of LIF expression was not statistically significant in CAFs cocultured with MDA-MB-231 (*p* < 0.419) after 24 h. However, after 48 h and 72 h, MCF7 and MDA-MB-231 increased LIF expression significantly, and this elevated expression was associated with time. Both cell lines, particularly MCF-7, significantly increased LIF expression in CAFs. Our in vitro study revealed that breast cancer cells stimulated CAFs to secrete LIF, which is in agreement with another study [32].

### 3.3. Inducing Stem-Cell-Like Properties in Breast Cancer Cells in the Presence of LIF

**Figure 3:**
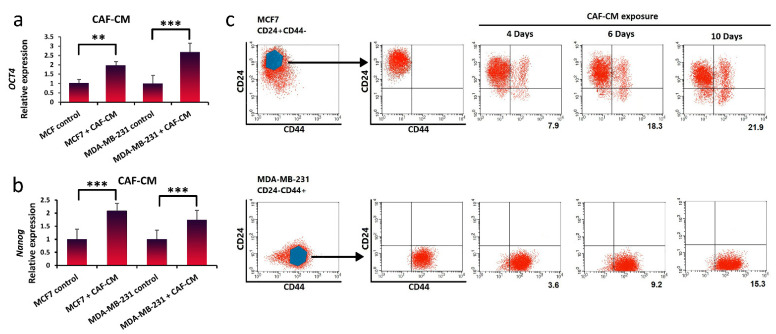
Breast cancer cells can be dedifferentiated and regain stem-cell-like properties through treatment with CAF-CM. (**a**,**b**) qRT-PCR analysis was used to measure *Nanog* and *Oct4* gene expression as cancer stem cell biomarkers in MDA-MB-231 and MCF7 cells cultured with or without CAF-CM after 10 days. Representative graphs of 3 independent experiments using the same CAF-CM are shown. The data show the mean ± S.D. and were analyzed using the Student’s *t*-test; ** *p* < 0.01 and *** *p* < 0.001. (**c**) Breast stem cell markers (CD24−/CD44+) were analyzed by flow cytometry in MCF7 (CD24+/CD44−) and MDA-MB-231 (CD24−/CD44+) cell lines after 4, 6, and 10 days. (Percentage of positive CD24−/CD44+ population is shown in numbers for MCF7, and the mean fluorescence intensity (MFI) is shown in numbers for MDA-MB-231).

After confirmation of LIF secretion by CAFs, to evaluate the effect of LIF cytokine on CSC plasticity, we treated breast cancer cells with CAF-CM. To confirm the LIF receptor expression in the breast cancer cell, qRT-PCR analysis was used (data are not shown). Breast cancer cells incubated with CAF-CM increased the expression levels of breast cancer stem cell markers *Nanog* and *Oct4* after 10 days (Figure 3a,b). The MCF7 cells exposed to CAF-CM showed a higher *Nanog* expression level compared to MDA-MB-231. It has been reported that this stemness marker, *Nanog*, correlates with carcinogenesis and poor clinical outcome, and patients with higher *Nanog* expression had a worse survival prognosis [33,34]. The MDA-MB-231 cells exposed to CAF-CM exhibited a higher Oct4 expression level compared to MCF7. It has been shown that Oct4-positive cases had a much lower overall survival rate, and *Oct4* enhanced EMT, contributing to the tumorigenesis and metastasis during in vitro study [34].

Moreover, following 4, 6, and 10 days of CAF-CM exposure of breast cancer cells, the expression of CD24/CD44 cell surface markers was analyzed by flow cytometry. We observed that breast stem cell markers (CD24−/CD44+) increased about 22% in MCF7 after 10 days (Figure 3c). Quantitatively, the mean fluorescence intensity (MFI) after CAF-CM exposure was significantly higher in MDA-MB-231 cells after 10 days. The results indicated that breast cancer cells (MCF7 and MDA-MB-231) incubated with CAF-CM could be dedifferentiated to reacquire cancer stem-like properties, with enhanced stemness marker (*Nanog* and *Oct4*) expression.

### 3.4. Promotion of Stem-Cell-like Properties in Breast Cancer Cells via Exogenous Exposure with LIF

**Figure 4:**
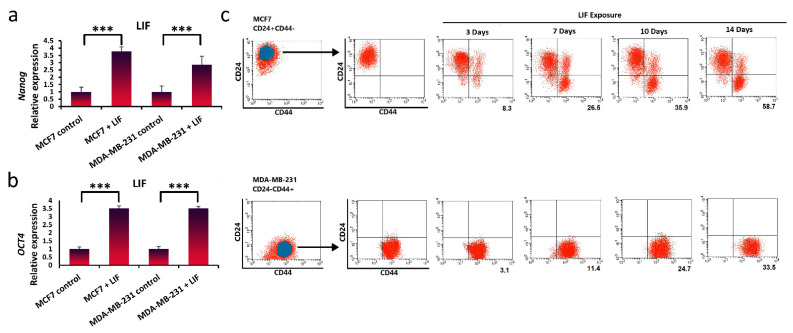
LIF exposure can dedifferentiate breast cancer cells and promote the acquisition of stem-cell-like properties. (**a**,**b**) qRT-PCR analysis was used to measure breast cancer stem cells gene expression (*Nanog* and *Oct4*) in MCF7 and MDA-MB-231 cells exposure to LIF after 14 days. Representative graphs of 3 independent experiments using the same CAF-CM are shown. The data show the mean ± S.D. and were analyzed using one-way ANOVA and the Student’s *t*-test; *** *p <* 0.001. (**c**) Breast stem cell markers (CD24−/CD44+) were analyzed by flow cytometry in MCF7 (CD24+/CD44−) and MDA-MB-231 (CD24−/CD44+) cell lines after 3, 7, 10, and 14 days. (Percentage of positive CD24−/CD44+ population is shown in numbers for MCF7, and the mean fluorescence intensity (MFI) is shown in numbers for MDA-MB-231).

To validate the impact of LIF on breast cancer stemness, we treated breast cancer cells with human LIF protein. Culturing breast cancer cells with LIF induced high levels of *Nanog* and *Oct4* expression, demonstrating characteristics of cancer cell stemness (Figure 4a,b). Furthermore, breast cancer cells MCF7 (CD24+/CD44−) and MDA-MB-231 (CD24−/CD44+) were continuously exposed to exogenous LIF and analyzed for CD24/CD44 expression variations over time. The continuous exposure of breast cancer cell populations to LIF resulted in an increase in the breast CSC marker relative to untreated cells in both populations. In MCF-7 cells, after 3 days of LIF exposure, CD24−/CD44+ expression was observed and continued until Day 14, which was about 59% converted to CD24−/CD44+ (Figure 4c). The quantity of MFI values obtained through flow cytometry was significantly increased by exposure to LIF in MDA-MB-231 cells after 14 days. However, more rapid conversion was seen in the MCF7 population.

### 3.5. Suppression of Breast Cancer Stemness via Blocking LIFR

**Figure 5:**
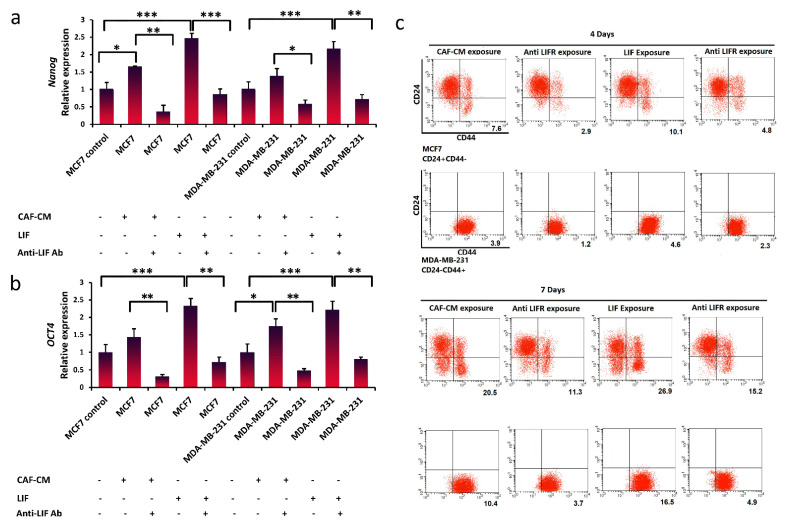
LIF/LIFR signaling pathway as a target to prevent breast cancer stemness. (**a**,**b**) qRT-PCR analysis of the stem cell marker *Nanog* and *Oct4* expression in MCF7 and MBA-MB-231 cells treated with CAF-CM or CAF-CM in combination with anti-LIFR Ab after 7 days. qRT-PCR analysis of *Nanog* and *Oct4* expression in MCF7 and MBA-MB-231 cells treated with LIF (25 ng ml) or LIF in combination with anti-LIFR Ab after 7 days. Representative graphs of 3 independent experiments using the same CAF-CM are shown. The data show the mean ± S.D. and were analyzed using the Student’s *t*-test; * *p* < 0.05, ** *p* < 0.01, and *** *p* < 0.001. (**c**) Breast stem cell markers (CD24−/CD44+) were analyzed by flow cytometry in MCF7 (CD24+/CD44−) and MDA-MB-231 (CD24−/CD44+) cells after 4 and 7 days. (Percentage of positive CD24−/CD44+ population is shown in numbers for MCF7, and the mean fluorescence intensity (MFI) is shown in numbers for MDA-MB-231).

To assess whether LIF signaling is a therapeutic pathway for the treatment of breast cancer by targeting breast CSCs, a special LIF receptor blockade antibody was utilized to inhibit this pathway in breast cancer cells. It was observed that *Nanog* and *Oct4* expression, which was substantially increased after LIF or CAF-CM treatment, was decreased following exposure to the LIFR inhibitor antibody in both breast cancer cell lines (Figure 5a,b). We further confirmed that the breast stem cell markers (CD24−/CD44+) were reduced following the blockade of LIFR signaling (Figure 5c). Using the LIFR inhibitor antibody revealed that blocking the LIFR is a sufficient way to inhibit the dedifferentiation of breast cancer cells.

## 4. Discussion

Several studies have revealed that the plasticity of cancer cells contributes to metastasis and tumor recurrence [35,36]. Recent studies have targeted how to eliminate cancer stem cells; however, the tumor microenvironment has played a significant role in specifying the malignant characteristics of cancer stem cells [37]. Determining whether the dedifferentiation of nontumorigenic cancer cells to cancer stem cells may occur in specific niches is very important. A number of studies have found that environmental factors regulate cell plasticity and induce dedifferentiation in normal somatic cells [38,39].

In this study, we investigated how a specific TME cytokine is associated with plasticity and CSC properties. Previous reports confirmed that hepatocyte growth factor and members of the IL-6 family stimulated the development of a CSC phenotype [40,41]. CAF-derived LIF induces nasopharyngeal carcinoma tumor growth [42], promotes the migration and invasion of tumor cells in oropharyngeal carcinomas and melanoma [43,44], stimulates angiogenesis in colon cancer [45], and induces chemoresistance in esophageal squamous cell carcinoma [46].

We investigated the LIF expression by CAFs since LIF secretion by CAFs in the breast tumor microenvironment is unclear. The current study shed light on the interaction of breast cancer cells and CAFs and revealed that breast cancer cells stimulated CAFs to produce LIF. CAFs in contact with breast cancer cells secrete LIF upon stimulation by specific factors expressed by the breast cancer cells. Although the mechanism of LIF expression by CAFs is still unknown, a recent study claimed that TGF-β could stimulate LIF secretion in tumor cells and fibroblasts [47]. Other studies have also found that fibroblast growth factor (FGF) and transforming growth factor (TGF-β) released from cancer cells could activate fibroblasts to release signaling molecules [48,49]. A more recent study has confirmed that during 24 h, MDA-MB-231 cells that are high-traction cells change collagen fibrils and alter mechanical characteristics near cells and lead to the detaching of collagen fibril from the surface [50]. This explains our results and why the level of LIF expression was not significant in CAFs cocultured with MDA-MB-231 after 24 h. Wang et al. reported that breast cancer cells recruit CAFs to regulate cellular behavior and remodel the extracellular matrix (ECM) by fibronectin (Fn) and collagen I (Col I) and facilitate tumor progression [51].

Fibroblasts play a significant role in supporting stem cell growth and the secretion of specific factors such as TGF-b1, LIF, and fibroblast growth factors that suppress differentiation through the WNT, NOTCH, Hedgehog, and EMT signaling pathways [52]. We indicated the significant role of the LIF/LIFR pathway as a regulator of breast cancer stemness. Our study showed that CAFs secrete LIF to stimulate the LIF receptor on breast cancer cells and thus activate the LIF/LIFR signaling pathway, which promotes breast cancer stemness in vitro. We observed that LIF expresses the stemness markers *Nanog* and *Oct4* in breast cancer cells. CAF-CM-treated cells were assessed for CD24/CD44 markers using flow cytometry. The percent change in the CSC marker (identified as CD24−/CD44+) was observed following treatment with CAF-CM compared to the untreated control. The capability of the LIF to induce cancer cell plasticity makes it a good choice for the treatment of breast cancer by neutralizing its function.

Therefore, we tested the impact of the exogenous LIF, and it altered the morphology of breast cancer cells to a cancer stem-cell-like phenotype, which was explained by the enhanced expression levels of dedifferentiation markers CD24−/CD44+. The supplementation of media with LIF for 2 weeks leads to cell-state changes from MCF7 and MDA-MB-231 to CD24−/CD44+ breast cancer stem cells. Our results are consistent with a variety of studies arguing that non-CSC can be transformed to CSC via changes between epithelial- and mesenchymal-like states by cytokines or chemotherapy [35,36,53]. Our examination validated the role of LIFR signaling in describing the cancer stemness characteristics in breast cancer cells.

On the other hand, the addition of the LIFR inhibitor antibody suppressed this alteration. Our data further indicate that blocking the LIF/LIFR signaling pathway could decrease cancer stemness in breast cancer cells, which points to a potential clinical application of targeted therapy using a LIFR inhibitor for breast cancer cells. Our findings indicated that LIF treatment transforms breast cancer cells into a stem-like state and acquires CSC properties. Consistent with the findings of Chen [54], we found that the differentiated cancer cells could dedifferentiate to cancer stem cells under the influence of the CAFs. Therefore, targeting cancer stem cells and targeting tumor microenvironment factors are crucial to inhibit cancer metastasis, drug resistance, and recurrence.

## 5. Conclusions

In conclusion, the results of this investigation bring new insights into the crosstalk between cancer stem cells and tumor microenvironment factors. We showed that breast cancer cells stimulated CAFs to secrete LIF, and in turn, the CAF-derived LIF regulates cancer stemness in breast cancer cells. Blockade of LIF signaling with the LIF receptor antagonist reverses these phenotypes and offers a possible therapeutic approach in breast cancer. Further, CAFs regulate cancer stem cells through the LIF/LIFR pathway and can be an anticancer therapy target. It should be highlighted that this study tested the two-dimensional interaction of CAFs and breast cancer cells, and their in vivo interaction remains to be further studied. In addition, future research should concentrate on the investigation of the effect of LIF on chemoresistance or drug-mediated resistance in breast cancer cells.

## Figures and Tables

**Table 1:** List of primer sequences used for quantitative real-time PCR.

Gene	Primer Sequence (5′-3′)	Amplicon Size	Annealing Temperature (°C)
*GAPDH*	Forward 5′-AGTCCACTGGCGTCTTCA-3′ Reverse 5′-GAGGCATTGCTGATGATCT-3′	163	58 55
*Oct4*	Forward 5′-GGGGGTTCTATTTGGGAAG-3′ Reverse 5′-TTGTCAGCTTCCTCCACCC-3′	126	57 59
*Nanog*	Forward 5′-CAGCTACAAACAGGTGAAGACC-3′ Reverse 5′-GGTGGTAGGAAGAGTAAAGGC-3′	146	62 61
*LIF*	Forward 5′-GCCCTCTTTATTCTCTATTACACAG-3′ Reverse 5′-ACACGACTATGCGGTACAGC-3′	151	63 60

## Abbreviations

CAF	cancer-associated fibroblast
CSC	cancer stem cells
EMT	epithelial–mesenchymal transition
LIF	Leukemia inhibitory factor
LIF-R	LIF receptor
TME	tumor microenvironment
JAk	Janus kinase
PI3K	phosphatidylinositol 3-kinase
STAT	signal transducer and activator of transcription
TGF	transforming growth factor
FGF	fibroblast growth factor
